# Comprehensive Genomic Analysis of Puerarin in Inhibiting Bladder Urothelial Carcinoma Cell Proliferation and Migration

**DOI:** 10.2174/1574892819666230908110107

**Published:** 2023-09-28

**Authors:** Yu-Yang Ma, Ge-jin Zhang, Peng-fei Liu, Ying Liu, Ji-cun Ding, Hao Xu, Lin Hao, Deng Pan, Hai-luo Wang, Jing-kai Wang, Peng Xu, Zhen-Duo Shi, Kun Pang

**Affiliations:** 1 Department of Urology, Xuzhou Central Hospital, Xuzhou Clinical School of Xuzhou Medical College, No.199, South Jiefang Road, Xuzhou, Jiangsu, China;; 2 Department of Urology, Graduate School, Bengbu Medical College, Building 1, Administration Building, 2600 Donghai Avenue, Bengbu City, Anhui Province, China;; 3 Department of Urology, Suqian Zhongwu Hospital. No. 3786, Development Avenue, Suqian Economic and Technological Development Zone, Suqian, China;; 4 Jiangsu Provincial Key Laboratory of Educational Big Data Science and Engineering, Jiangsu Normal University, 101 Shanghai Road, Tongshan, Xuzhou, 221116, China;; 5 School of Mathematics and Statistics and Research Institute of Mathematical Sciences (RIMS), Jiangsu Normal University, 101 Shanghai Road, Tongshan, Xuzhou, 221116, China;; 6 Department of Central Laboratory, Xuzhou Central Hospital, Xuzhou Clinical School of Xuzhou Medical College. No.199, South Jiefang Road, Xuzhou, Jiangsu, China;; 7 Department of Burn and Plastic Surgery, Xuzhou First People's Hospital, No. 269, Daxue Road, Tongshan District, Xuzhou, Jiangsu, China;; 8 Graduate School, Jiangsu University, 301 Xuefu Road, Zhenjiang, Jiangsu Province, 212013, China

**Keywords:** Puerarin, bladder urothelial carcinoma (BUC), proliferation, migration, protein-protein interaction, bioinformatics

## Abstract

**Background:**

Bladder urothelial carcinoma (BUC) ranks second in the incidence of urogenital system tumors, and the treatment of BUC needs to be improved. Puerarin, a traditional Chinese medicine (TCM), has been shown to have various effects such as anti-cancer effects, the promotion of angiogenesis, and anti-inflammation. This study investigates the effects of puerarin on BUC and its molecular mechanisms.

**Methods:**

Through GeneChip experiments, we obtained differentially expressed genes (DEGs) and analyzed these DEGs using the Ingenuity® Pathway Analysis (IPA®), Kyoto Encyclopedia of Genes and Genomes (KEGG) and Gene Ontology (GO) pathway enrichment analyses. The Cell Counting Kit 8 (CCK8) assay was used to verify the inhibitory effect of puerarin on the proliferation of BUC T24 cells. String combined with Cytoscape® was used to create the Protein-Protein Interaction (PPI) network, and the MCC algorithm in cytoHubba plugin was used to screen key genes. Gene Set Enrichment Analysis (GSEA®) was used to verify the correlation between key genes and cell proliferation.

**Results:**

A total of 1617 DEGs were obtained by GeneChip. Based on the DEGs, the IPA® and pathway enrichment analysis showed they were mainly enriched in cancer cell proliferation and migration. CCK8 experiments proved that puerarin inhibited the proliferation of BUC T24 cells, and its IC50 at 48 hours was 218µmol/L. Through PPI and related algorithms, 7 key genes were obtained: ITGA1, LAMA3, LAMB3, LAMA4, PAK2, DMD, and UTRN. GSEA showed that these key genes were highly correlated with BUC cell proliferation. Survival curves showed that ITGA1 upregulation was associated with poor prognosis of BUC patients.

**Conclusion:**

Our findings support the potential antitumor activity of puerarin in BUC. To the best of our knowledge, bioinformatics investigation suggests that puerarin demonstrates anticancer mechanisms *via* the upregulation of ITGA1, LAMA3 and 4, LAMB3, PAK2, DMD, and UTRN, all of which are involved in the proliferation and migration of bladder urothelial cancer cells.

## 1 INTRODUCTION

A study by the American Cancer Society in 2021 estimated that bladder urothelial cancer (BUC) had the fourth-highest incidence and eighth-highest mortality among men in the United States [1]. Due to high recurrence, BUC carries a large social burden, with over 430,000 men and women diagnosed worldwide yearly [2]. BUC is a heterogeneous disease associated with various clinical outcomes, and conventional chemotherapy treatments are unsuccessful in curbing chemoresistance and BUC progression while having several adverse side effects [3, 4]. Therefore, finding a therapy to improve patient outcomes and reduce side effects and drug resistance is necessary.

Traditional Chinese medicine (TCM) advocates the treatment of diseases in the form of complexes, and multiple TCM complexes have been confirmed to have anticancer effects [5, 6]. Lately, a variety of TCM monomers, such as phenylpropanoids alkaloids, and flavonoids have been confirmed to have anti-tumor effects [7-9]. TCM has previously been used for cancer treatment. It can not only limit the occurrence of tumors but also reduce the side effects of chemoradiotherapy due to its multi-component, and multi-target characteristics. In addition, TCM also has a positive effect in enhancing host immune function, prolonging patient survival, and reducing the risk of some chronic lung diseases [10]. In clinical practice, TCM is commonly used as adjuvant chemotherapeutic drugs to enhance the efficacy of chemotherapeutic drugs and improve the quality of life of patients [11].

Puerarin is an isoflavone derivative isolated from the TCM Kudzu root that has effects on multiple systems of the human body. An *in*-*vitro* study found that puerarin alleviated nephrotoxicity by regulating Toll-like receptor 4 (TLR4)/ Nuclear factor-kappaB (NF-κB) signaling pathway [12]. The T24 cell line belongs to that of metastatic urothelial carcinoma of the bladder and has a good representative role in advanced refractory bladder malignancies. Liu *et al*. found that puerarin could inhibit BUC cell proliferation, promote apoptosis, and block the NF-κB signaling pathway by upregulating the expression of mir-16 in T24 cells [13]. This study predicts that puerarin may impart favorable outcome in BUC patients. It has been reported that puerarin has the capability of lowering blood pressure, reducing myocardial oxygen consumption, dilating coronary vessels, protecting the liver, controlling blood sugar, and suppressing cancers and ischemia-reperfusion injury [14]. Most of the studies on the mechanism of action of puerarin in inhibiting BUC were not based on high-throughput basic experimental studies and their findings showed that puerarin restrained BUC cells by regulating The mechanistic target of rapamycin (mTOR) /p70S6K [15], circ_0020394/miR-328-3p/nuclear receptor-binding protein 1 (NRBP1) [16], silent information regulator sirtuin 1 (SIRT1)/p53 [17], and miR-16 [13]. Although puerarin has been partially studied in BUC, the mechanism by which puerarin inhibits BUC cells remains to be explored. In this study, we used high-throughput and bioinformatics analysis to predict how puerarin inhibits BUC. We hope to provide some directions for subsequent studies.

## 2 MATERIALS AND METHODS

### 2.1 Materials and Reagents

Puerarin was purchased from Solarbio Life Sciences Co., Ltd, Beijing, China, and was dissolved in Dimethyl sulfoxide (DMSO) to prepare an 800 µM stock solution stored at -20°C before use. The bladder urothelial cancer T24 cell line was purchased from the Cell Resource Center, Shanghai Institutes for Biological Sciences at the Chinese Academy of Sciences. An Agilent RNA 6000 Nano Kit was purchased from Agilent Technologies (Van Nuys, CA); an *in vitro* reverse transcription kit, GeneChip 3′ IVT Express Kit (US Affymetrix) for RNA purification, and a GeneChip Hybridization Wash and Stain Kit were purchased from Affymetrix.

### 2.2 Cell Culture

The BUC T24 cells were routinely cultured in a 1640 Dulbecco's modified Eagle medium (DMEM) containing 10% FBS in a 5%-CO^2^ saturated humidity incubator at 37°C. When the cells were cultured in a 10 cm dish until the confluence reached 80% - 90%, the culture medium was poured off and T24 cells were washed twice with 3 ml D-HANKS. Then, 1 ml of trypsin solution was added and mixed well to digest the T24 cells. When cells fell off in sheets, 3 ml of 1640 DMEM culture medium containing 10% FBS was added to terminate the digestion. The cells were blown to form single cells and then centrifuged at 1000 rpm, 4°C, for 5 minutes. After that, the supernatant was discarded and T24 cells were incubated in new Petri dishes and the complete medium was added.

### 2.3 Cell Viability Assay and IC_50_

Cell viability assay with Cell Counting Kit-8 (CCK8) was used to quantify T24 cell viability. The T24 cells were seeded onto 96-well plates at a density of 4000 cells/well for 48 h and then incubated with RPMI-1640 medium containing various dilutions of puerarin (12.5, 25.0, 50.0, 100.0, 200.0, 300.0, 400.0, 600.0 and 800.0µmol/l) and negative control (medium only) at 37°C in a 5% CO^2^ humidified atmosphere for 48 h. Following incubation for the indicated times, 10 µl CCK8 solution was added to each well and incubated for 2 h at 37°C to examine the effect of puerarin on BUC cell proliferation. The measurement of cell viability and IC_50_ values *via* relative optical density was set at 450 nm. Every experiment was performed in triplicate.

### 2.4 Affymetrix Gene Expression Microarrays

RNA was tested for degradation and RNA concentration was determined using an Agilent ND-1000 before labeling the RNA. Samples were labeled using the Agilent Quick Amp Labeling kit, and hybridization experiments were performed using Agilent SureHyb. The chips were washed and then scanned by using an Agilent DNA Microarray Scanner. Chip probe signal values were acquired using Agilent Feature Extraction software (v11.0.0.1). Chip normalization and raw data were performed using Agilent GeneSpring GX v12.1 software.

We compared and analyzed the Affymetrix gene expression profiling microarray chip data of the puerarin treatment group and normal control group in T24 cells, and absolute expression signals as well as log transformation of fold change using base 2. Screening criteria were required to satisfy log_2_FC > 2 and a P-value < 0.05. Through the criteria, we obtained differentially expressed genes (DEGs).

### 2.5 Bioinformatic Analyses

The bioinformatic analyses were performed to find thepotential related pathways and genes related to the inhibitory effect of puerarin on T24 cells. The analyses primarily consisted of enrichment analyses of diseases and pathways, protein-protein interactions (PPI), gene set enrichment analysis and survival curves.

#### 2.5.1 Volcano Plot

The volcano plot was made by R-package plot in Rstudio (2022.02.0) and was used to show the distribution of all genes, especially DEGs. The DEGs were defined as -1 < log2[Fold Change] < 1 and P-value < 0.05.

#### 2.5.2 Ingenuity^®^ Pathway Analysis

According to the above screening method, the genes were divided into two groups of up- and down-regulated genes and introduced into Ingenuity^®^ pathway analysis (IPA^®^) which mainly includes four functions: diseases and functions, canonical pathway, upstream analysis and regulator effects. Through these functions, we can get which pathways and diseases the DEGs were enriched in and the upstream regulatory factors and relative networks.

#### 2.5.3 Pathway Enrichment Analysis

Metascape^®^ (http://metascape.org) is one of the most commonly used bioinformatics websites that can make pathway enrichment analyses and transcription factor predictions [18]. KEGG and GO pathway analysis, which is respectively based on the Kyoto Encyclopedia of Genes and Genomes (KEGG, https://www.genome.jp/kegg/) and Gene Ontology (GO, http://geneontology.org), are the two most familiar database that are accepted. Adobe Illustrator 20 and R-package “enrichplot,” “ggplot2,” “clusterProfiler,” and “GOplot” in Rstudio software were performed for pathway enrichment analysis and graph modification.

#### 2.5.4 Protein-protein Interactions

Protein-protein interactions (PPIs) were made by String (version 11.5, https://cn.string-db.org/), which aims to collect, score and integrate all publicly available sources of PPI information, and to complement these with computational predictions [19]. Cytoscape^®^ (V3.9.1) is a software performed to further optimize PPIs and obtain key genes through algorithm MCC in plug-in cytohubba. GeneMANIA (http://genemania.org) was used to construct and analyze a functional network of key genes and their co-expressed genes.

#### 2.5.5 Survival Curves

Gene expression profiling interactive analysis (GEPIA^®^, http://gepia.cancer-pku.cn/) is a web server for cancer and normal gene expression profiling and interactive analyses based on TCGA and GTEx data. It provides key interactive and customizable functions, including differential expression analysis, profiling plotting, correlation analysis, patient survival analysis, similar gene detection and dimensionality reduction analysis [20]. The Methods and Cut Off were respectively chosen Overall Survival and Quartile. Both Hazards Ratio (HR) and 95% Confidence Interval were chosen to Yes, and Datasets Selection was set to BLCA.

#### 2.5.6 Gene Set Enrichment Analysis

Gene Set Enrichment Analysis (GSEA**^®^**, https://www.gsea-msigdb.org/gsea/index.jsp) is a computational method that determines whether **2** priori-defined set of genes shows statistical significance, and concordant differences between two biological states [21]. The genome expression data was downloaded from GDC (https://portal.gdc.cancer.gov/). Gene set data named “GOBP_NEGATIVE_REGULATION_OF_EP ITHELIAL_CELL_PROLIFERATION” whose brief description was “Any process that stops, prevents or reduces the rate or extent of epithelial cell proliferation” was downloaded from GSEA^®^. Here, GSEA was performed to identify whether a certain predefined gene set was enriched in the expected relevant pathway by using GSEA_4.2.3. “collapse/remap to gene symbols” was set to “No_Collapse” and “Permutation type” was set to “Phenotype”.

### 2.6 Statistical Analysis

Statistical analyses were performed by using IBM SPSS Statistics 27. Student’s t-test was performed to compare the differences between the two groups. One-way ANOVA was performed to analyze the differences among multiple groups. Kaplan-Meier curves and log-rank tests were used for plotting survival curves. Statistical significance was set at *p* < 0.05.

## 3 RESULTS

### 3.1 Puerarin Inhibited the Viability of T24 Cells

**Fig. (1):**
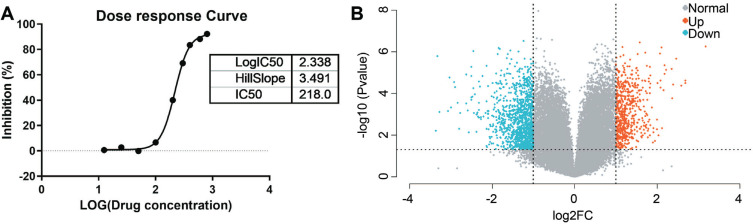
The DEGs. (**A**) The dose-response curve of puerarin on T24 cells demonstrates the IC_50_ was 218 µmol/L. (**B**) The volcano plot of all genes contained in Affymetrix^®^ gene expression microarrays. The blue color on the left side represents 1053 downregulated genes, the red color on the right side represents 564 upregulated genes.

To evaluate the effect of puerarin on cell viability, T24 cells were incubated with puerarin at a concentration of 0, 12.5, 25, 50, 100, 200, 300, 400, 600, and 800 µmol/L for 48 h. The results demonstrated a significant (*p* < 0.05) increase in cell death that occurred in a dose-dependent manner upon incubation with increasing concentrations of puerarin (100-800 µM) (Fig. **1A**). The highest cell death was found at a puerarin concentration of 218 µM.

### 3.2 The DEGs in Cells Treated with or without Puerarin

To explore the mechanism by which puerarin inhibits BUC T24 cells, the whole genome Affymetrix microarray chip hybridization was adopted to discover the gene expression of T24 cells between the control groups and puerarin-treated groups. The volcano plot showed that 1617 differentially expressed genes (DEGs), including 564 upregulated and 1053 downregulated genes, were identified (Fig. **1B**). The details of DEGs are listed in Table **S1**. In addition, the flowchart of the study based on DEGs is shown in Fig. (**2**).

### 3.3 DEGs were Associated with Cell Proliferation and Migration

The DEGs were analyzed by Ingenuity^®^ pathway analysis (IPA^®^), which includes several functions such as diseases and functions, canonical pathway, upstream analysis and regulator effects. Through these functions, we can know which pathways and diseases the DEGs are enriched with and the upstream regulatory factors and relative networks.

#### 3.3.1 Diseases and Functions

Disease and function analysis with IPA^®^ assessed the positive association between puerarin and other diseases or functions. As shown in Fig. (**3A**), the results showed that DEGs were mostly enriched in some physiological and pathological conditions such as Cancer, Organismal Injury and Abnormalities, Cellular Movement, Cell Death and Survival, Tissue Morphology and a series of Systemic diseases. The information related to Diseases and Functions is shown in Table **S2**. Among these diseases and functions associated with malignant tumors were Cancer, Cellular Movement, Cell Death and Survival.

#### 3.3.2 Canonical Pathway Analysis

Canonical pathway analysis of IPA^®^ elucidated on which pathways DEGs are enriched predominantly. A total of 55 classical pathways were obtained by the pathway analysis (*p* < 0.05). The top 20 pathways ranked by z-score were shown in Fig. (**3B**) and there were 5 pathways that were highly related to tumorigenesis. These pathways include DNA Double-Strand Break Repair by Homologous Recombination (-log *p* = 4.06), Molecular Mechanisms of Cancer (-log *p* = 2.88), HIF1α Signaling (-*log p* = 2.84), Role of BRCA1 in DNA Damage Response (-log *p* = 2.59) and Wound Healing Signaling Pathway (-log *p* = 2.35). The information related to the Canonical pathway is listed in Table **S3**.

#### 3.3.3 Upstream Analysis

**Fig. (4):**
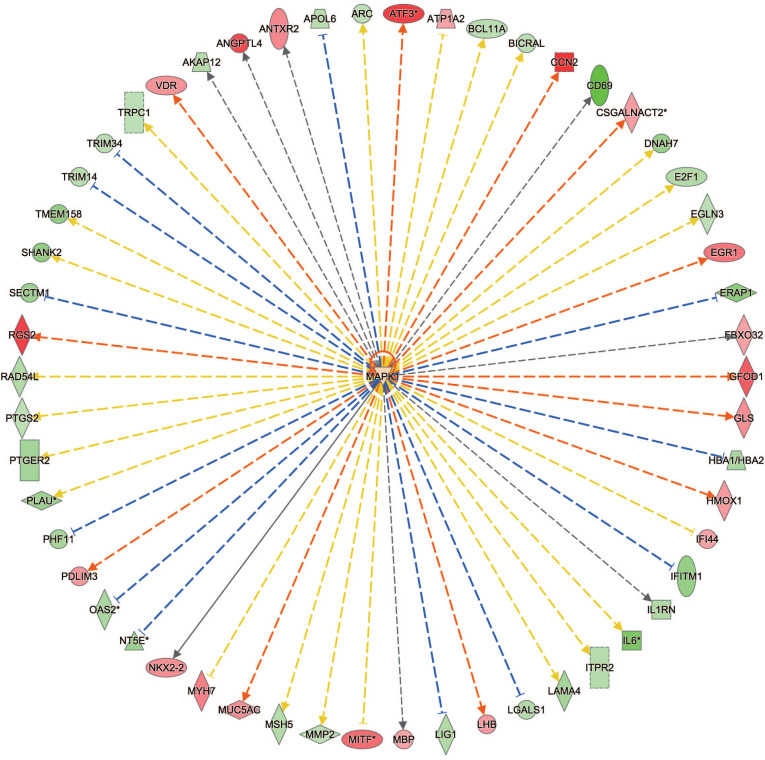
Upstream regulator (MAPK1) by IPA^®^. Gene networks of upstream regulators related to tumorigenesis: MAPK1 network. The color shade and font size of a gene tag are proportional to the absolute value of its log (FC), with red representing its log (FC) as positive and green representing its log (FC) as negative.

Upstream analysis was able to predict the upstream regulatory factors of genes from DEGs. These factors could be chemical drugs, kinase, cytokine, enzyme, transcription regulator, complex, or others. Meanwhile, Upstream analysis generated many relationship graphs, each showing which genes from the DEGs are affected by an upstream regulator, with red representing genes up-regulated and green representing genes down-regulated. The predictive interactions were supported by literature based on the Ingenuity Pathway Knowledge Base (IPKB) [22]. An overlap P-value was computed based on the significant overlap between genes in the DEGs, and the z-score was used to make predictions. As presented in Table **1**, IPA^®^ predicted the most probable upstream regulators including MAPK1 (kinase, z-score = 0.368, *p*-value = 1.57E-08, Fig. **4**), ERBB2 (kinase, z-score = -1.864, *p*-value = 3.02E-08), VEGF (group, z-score = -1.07, p-value = 2.24E-0), IKBKB (kinase, z-score = -2.065, *p*-value = 8.40E-08), ESR2(ligand-dependent nuclear receptor, z-score = -2.371, *p*-value = 3.67E-07), and TNF (cytokine, z-score = -1.428, *p*-value = 4.24E-07). The full information is listed in Table **S4**.

#### 3.3.4 Regulator Effects

The regulatory effect network diagram shows the interactions between genes and regulators and functions in IPKB. The consistency score is a measure of the causal consistency and tight link between upstream regulatory factors and disease and function in IPKB. Usually, the more accurate the results of regulation prediction, the higher the consistency score is, and the more accurate the results of the prediction of the regulatory effects. From the results shown in Table **2**, regulators affected diseases and functions consisting of Vascularization, Migration of cells, Morbidity and mortality, Cell viability, hypoplasia, vascular tumor and others. Obviously, they were predominantly related to angiogenesis and tumorigenesis. The details are listed in Table **S5**.

### 3.4 DEGs were Enriched in Proliferation and Migration

The results of KEGG and GO pathway enrichment analyses were obtained from Metascape^®^, which is a website used to make a comprehensive informatics analysis.

#### 3.4.1 KEGG Enrichment Analysis

**Fig. (5):**
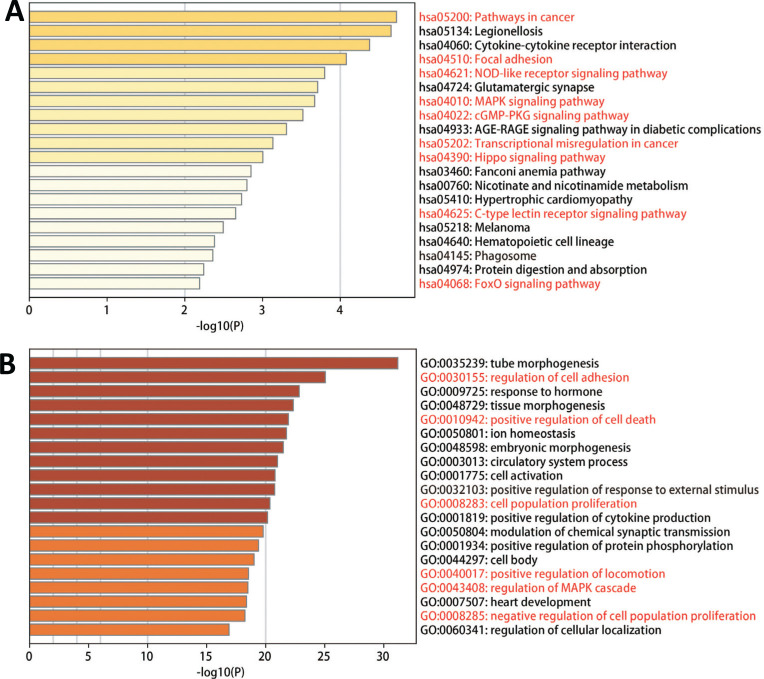
Pathway enrichment analyses by metascape^®^ showed that DEGs were enriched in pathways related to cancer, cell proliferation and migration. (**A**) The top 20 results of the KEGG pathway analysis ranked by -log *p*. (**B**) The top 20 results of biological processes ranked by -log *p*.

Among the KEGG pathways, there were almost half of the pathways were tightly correlated with cancer and cell adhesion. These pathways included Pathways in cancer, Focal adhesion, NOD-like receptor signaling pathway, MAPK Signaling pathway, cGMP-PKG signaling pathway, Transcriptional misregulation in cancer, Hippo signaling pathway, C-type lectin receptor signaling pathway, and FoxO signaling pathway (Fig. **5A**).

#### 3.4.2 GO Biological Process Analysis

The GO analyses in Fig. (**5B**) showed that six pathways were correlated with cell proliferation and locomotion. These pathways included regulation of cell adhesion, positive regulation of cell death, cell population proliferation, positive regulation of locomotion, regulation of MAPK cascade and negative regulation of cell population proliferation.

### 3.5 Protein-protein Interactions and Key Genes: ITGA1, LAMA3, LAMB3, LAMA4, PAK2, DMD and UTRN

To further understand the relationship among the DEGs, a website named String was used to obtain the list of PPIs. The minimum required interaction score was set to high confidence (0.900), and disconnected nodes in the network were hidden. Fig. (**6**) shows the Top 50 genes of MCC through CytoHubba in Cytoscape^®^. The shade of circular color is directly proportional to its importance. The redder the color, the more important. The top 50 scores by MCC are listed in Table **S5** and Fig. (**6**) elucidated that ITGA1, LAMA3, LAMB3, LAMA4, PAK2, DMD and UTRN were the key genes of puerarin on BUC T24 cells. In addition, GeneChip analysis demonstrated that ITGA1, LAMA3, LAMB3, LAMA4 and PAK2 were down-regulated whereas DMD and UTRN were up-regulated.

**Fig. (6):**
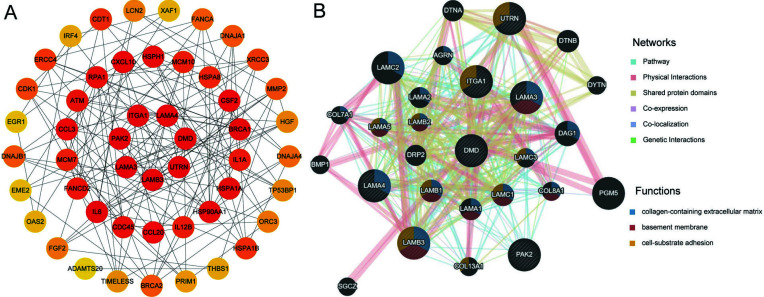
Protein-protein interaction network by String^®^ and Cytoscape^®^. The PPI network of the top 50 genes in MCC and the color depth of the circle are proportional to the importance of corresponding genes.

To explore the in-depth relationships, the 7 key genes and their co-expression genes were analyzed using GeneMANIA. GeneMANIA constructed a network of 27 genes, including 20 related genes. A total of 7 hub genes showed a complex PPI network with a Pathway of 35.45%, Physical Interactions of 24.83%, shared protein domains of 24.81%, and Co-expression of 10.81%. The functional information in the network revealed the importance of collagen-containing extracellular matrix, basement membrane, cell-substrate adhesion, *etc*. (Fig. **6B**). The complete function information is seen in Table **S7**.

### 3.6 Gene Set Enrichment Analysis (GSEA^®^) on Key Genes that Correlate with Cell Proliferation

**Fig. (7):**
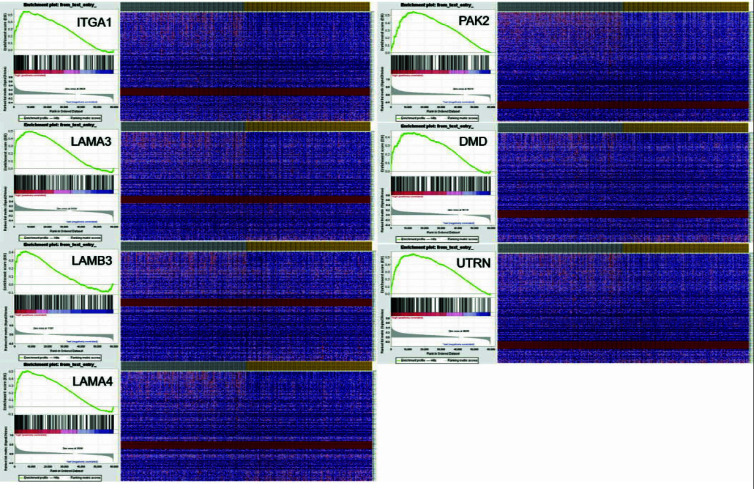
GSEA revealed that key genes were enriched in proliferation. Heatmap of gene expression abundance. Graph of GSEA enrichment analysis results.

GSEA was performed to observe the pertinence between key genes and cell proliferation. Gene expression and cell proliferation data were downloaded from GDC and GSEA^®^. The results elucidated that key genes were significantly enriched in cell proliferation pathways (Fig. **7**) and heatmap showed the expression of genes associated with proliferation (Fig. **7**). Normalized Enrichment Score (NES) and False Discovery Rate (FDR) were listed in Table **3**. In consideration of previous research results, puerarin inhibits the proliferation of BUC cells.

### 3.7 Survival Curve by Gene Expression Profiling Interactive Analysis (GEPIA®): ITGA1 was an Effective Target for Treatment

To verify whether patient survival is related to these genes gained from PPIs, the patient overall survival analysis in GEPIA^®^ was used to make a series of survival curves. The results in Fig. (**8**) showed that high expression of ITGA1 was associated with low survival in BUC patients. Additionally, both *p*-values of LAMA4 and DMD were less than 0.1, and they may be potentially effective therapeutic targets.

#### 3.7.1 Separate Pathway Enrichment of Up and Downregulated Genes

**Fig. (9):**
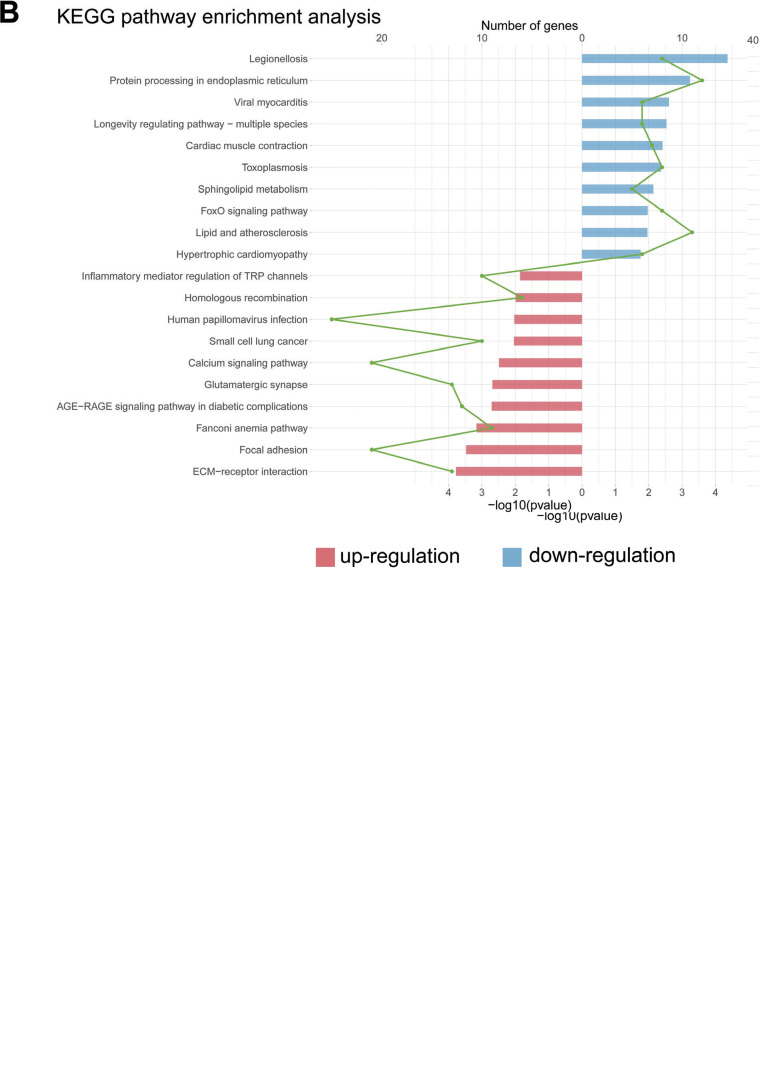
**(A, B).** Separate pathway enrichment of up- and downregulated genes.

The upregulated genes in GO pathways were enriched in protein refolding, inclusion body assembly, actomyosin, aggresome, unfolded protein binding and ATP-dependent protein folding chaperone; the downregulated genes in GO pathways were enriched in calcium ion transmembrane import into the cytosol, positive regulation of tumor necrosis factor superfamily cytokine production, nuclear chromosome, basement membrane, interleukin-6 receptor binding, and growth factor receptor binding (Fig. **9A**). The upregulated genes in KEGG pathways were enriched in legionellosis and protein processing in the endoplasmic reticulum; the downregulated genes in KEGG pathways were enriched in ECM-receptor interaction and focal adhesion (Fig. **9B**).

## 4 DISCUSSION

In this study, we investigated the effect of puerarin on the BUC T24 cell line using bioinformatics analyses. Our findings revealed that puerarin may inhibit the proliferation and migration of T24 cells through seven key genes, namely, ITGA1, LAMA3, LAMB3, LAMA4, PAK2, DMD, and UTRN. Further studies showed that ITGA1 upregulation was associated with poor prognosis of BUC patients.

The CCK8 assay showed that puerarin had a great inhibitory effect on the viability of T24 cells and the half-maximal inhibitory concentration was 218 µM. Ye *et al*. found that the inhibitory effect of puerarin on BUC T24 cells was only observed when the concentration of puerarin was greater than 100 µg/ml, *i.e*. 240 µM [17], which was similar to our study. Additionally, a study by Du *et al*. found significant cell viability inhibition by puerarin on the BUC T24 cells when its concentration was 100 mg/ml [16]. Although puerarin had a more efficient viability inhibition of T24 cells at a concentration of 100 µg/ml, the study by Liu *et al*. concluded that the half-inhibitory concentration of puerarin on T24 cells was around 50 µg/ml [13]. It is well believed that puerarin has a great anticancer effect and the optimal inhibitory concentration is 218 µM, though a few studies show discordance.

Puerarin is a TCM monomer extracted from the Kudzu root, which has a wide range of effects in a variety of systems, including the cardiovascular system, and can be used to treat a variety of diseases, including atherosclerosis, cardiomegaly, heart failure, and diabetic cardiovascular complications [23]. A meta-analysis studying the efficacy and safety of puerarin injection in curing acute ischemic stroke by Zheng *et al*. showed that no significant adverse effects were observed when 600 mg of puerarin was administered intravenously [24], which was also supported by a study by Liu *et al*. [25]. The normal blood volume of the human body is considered to be 4000-5000 ml, and assuming that the blood volume of the subject is 4500 ml, the blood concentration of puerarin after an injection of 600 mg is about 0.133 mg/ml, *i.e.*, 320 μM. Thus, we think that the concentration of 218 μM puerarin has no significant negative effects on healthy cells and may be suitable for the human body.

Research on puerarin in BUC is limited, and existing studies have focused on the inhibitory effects of puerarin on the proliferative activity, migration and invasion of BUC cells with traditional methodology [16]. In this study, we explore the effect of puerarin on BUC cells comprehensively in a high-throughput way combined with bioinformatics analyses. As shown by the results, puerarin inhibited the proliferation of BUC T24 cells and also had an inhibitory effect on the migration and invasion of cells, which is consistent with previous studies [16]. In addition, IPA^®^ analysis showed that puerarin mainly mediated DNA damage in the nucleus of BUC cells, and biological target prediction analysis revealed that the possible key targets of puerarin were ITGA1, LAMA3, LAMB3, LAMA4, PAK2, DMD, and UTRN.

Integrins are transmembrane heterodimers presented on the surface of multitudinous cells and are composed of α and β subunits. Its function depends on calcium or magnesium ions. The widely known function of integrins is mediating mutual recognition and adhesion between cells and cells as well as between cells and extracellular matrix. The ITGA1 gene encodes the α1 subunit of the integrin receptor, which heterodimerizes with the β1 subunit to form cell surface receptors for collagen and laminin, promoting tumorigenicity in malignant tumors [23]. Based on the hub-gene analysis and related studies, we suggested that puerarin may have a prominent effect in inhibiting tumor migration and that this effect is related to the inhibition of ITGA1 expression.

Laminins, a family of extracellular matrix glycoproteins, are the major noncollagenous constituent of basement membranes and they have been implicated in a wide variety of biological processes, including cell adhesion, differentiation, migration, signaling, neurite outgrowth and tumor cell metastasis [24]. LAMA located in chromosome 6q21, encodes the alpha chain isoform laminin, alpha 4 [25]. LAMA4 is a cancer-treatment target and high expression of LAMA4 is associated with various malignant tumors such as hematologic neoplasm [26], digestive system neoplasms [27, 28] and genitourinary neoplasms [29, 30]. LAMA4 is a cancer-associated gene and a very promising malignant tumor therapeutic target.

In addition, high expression of other key genes like PAK2 [31], LAMB3 [32] and LAMA3 [33] is also associated with malignant tumors. It is well-known that DMD gene mutation can cause Duchenne and Becker muscular dystrophy. However, increasing evidence shows that DMD is associated with cancer development [34]. DMD and UTRN negatively correlate with tumorigenesis [35, 36].

Although important discoveries were revealed by these studies, there are also limitations. First, it is the selected DEGs rather than the total genes that were included in the analysis, which may lead to deviations in the study results from the actual situation. Second, in this study, only some mechanisms were selected for detailed validation and exploration, and it did not prove that all study predictions were true and reliable. Lastly, we predicted the upstream regulators, comprehensive action pathways and targets of puerarin based on bioinformatics, but did not rule out certain errors with the actual situation, which requires further experimental verification.

This comprehensive prospective prediction study helps with the understanding of the cancer suppression mechanisms of puerarin and the role of puerarin in some systemic diseases. Overall, this study can provide a comprehensive, multi-faceted research direction for the subsequent study of puerarin in cancer.

## CONCLUSION

Our findings support the potential antitumor activity of puerarin in BUC. To the best of our knowledge, bioinformatics investigation suggests that puerarin demonstrates anticancer mechanisms *via* the upregulation of ITGA1, LAMA3 and 4, LAMB3, PAK2, DMD, and UTRN, all of which are involved in the proliferation and migration of bladder urothelial cancer cells.

## Figures and Tables

**Fig. (2):**
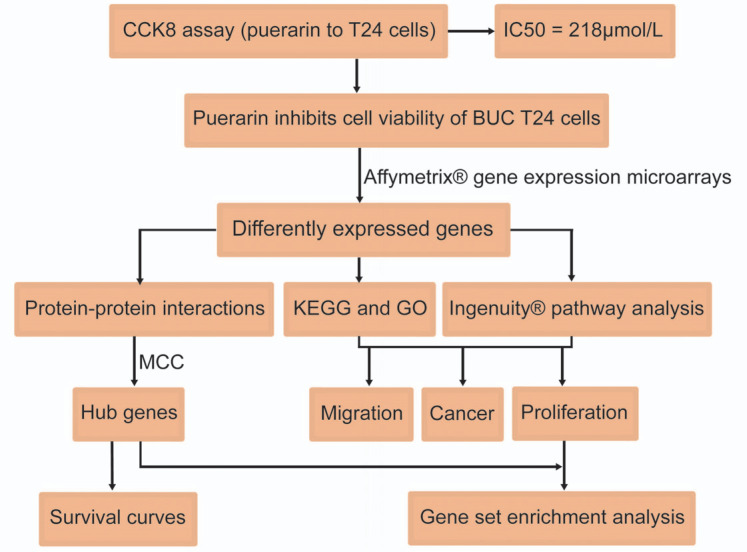
Flowchart of this study. The flowchart elucidates the research strategy of this study.

**Fig. (3):**
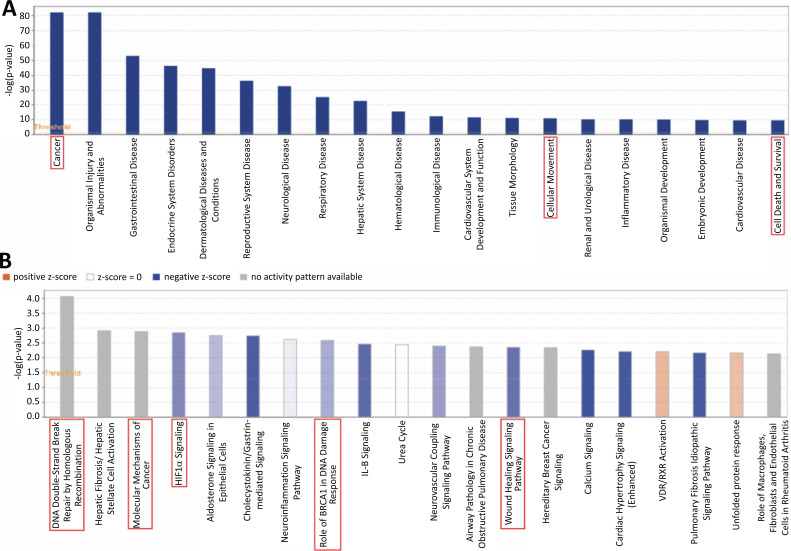
Diseases and functions and canonical pathway analysis by IPA^®^. (**A**) The top 20 cluster of the differential genes in the disease and functional categories, which were ranked by -log (*p*-value). (**B**) The top 20 pathways in canonical pathway analysis by IPA^®^.

**Fig. (8):**
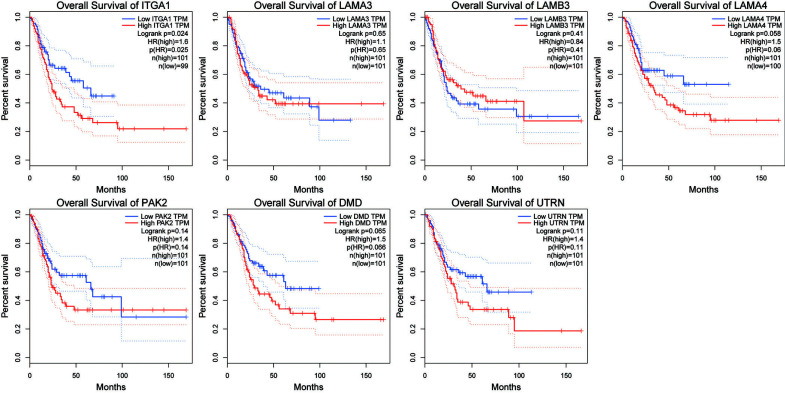
Survival curves by GEPIA^®^. The overall survival curves of key genes show that BUC patients with high expression of ITGA1 was significantly related to low survival and high expression of LAMA4 and DMD was very likely associated with low survival.

**Table 1:** Upstream regulator by IPA^®^. Upstream regulators mainly consist of MAPK1, ERBB2, IKBKB, VEGF, ESR2 and TNF.

**Upstream Regulator**	**Molecule Type**	**Z-score**	***p*-value**
Tetradecanoylphorbol acetate	Chemical drug	-0.747	7.31E-10
Dexamethasone	Chemical drug	0.836	3.28E-09
MAPK1	Kinase	0.368	1.57E-08
ERBB2	Kinase	-1.864	3.02E-08
Progesterone	Chemical - endogenous mammalian	-0.327	5.64E-08
IKBKB	Kinase	-2.065	8.40E-08
Salmonella enterica serotype abortus equi lipopolysaccharide	Chemical toxicant	1.245	1.29E-07
Bortezomib	Chemical drug	1.874	1.84E-07
VEGF	Group	-1.07	2.24E-07
Tributyrin	Chemical drug	0.383	2.47E-07
ESR2	Ligand-dependent nuclear receptor	-2.371	3.67E-07
Trichostatin A	Chemical drug	1.504	3.68E-07
CG	Complex	-0.357	4.01E-07
TNF	Cytokine	-1.428	4.24E-07
TREM1	Transmembrane receptor	-0.757	5.16E-07

**Table 2:** Regulator effect by IPA^®^. The result of regulator effect network analysis showed that the regulators mainly affected angiogenesis and tumorigenesis.

**Genes**	**NES**	**FDR**
ITGA1	2.295842	< 0.001
LAMA3	2.084553	< 0.001
LAMB3	1.759958	0.002114
LAMA4	2.148072	< 0.001
PAK2	2.412002	< 0.001
DMD	1.939933	< 0.001
UTRN	2.333468	< 0.001

**Table 3:** NES and FDR of key genes by GSEA^®^. NES of all key genes were more than 1.50 and FDR were less than 0.01.

**Regulators**	**Target Molecules in Dataset**	**Diseases & Functions**	**Consistency Score**
CSF, CX3CR1, FBXO32, HAND2, HDAC2, HEY2, KLF6	ATF3, BRCA1, CACNA1C, CSF2, CXCR2, E2F1, FGF2, FGF9, GADD45A, GLI1, HMOX1, HSPB7, IL1A, IL1RN, IL6, IL6ST, ITGB8, KCNJ2, LIG1, PLAU, PROX1, PTGS2, SLC2A2, TPM1	Activation of hepatic stellate cells, cell proliferation of fibroblasts, congestive heart failure, proliferation of liver cells, vascularization	7.144
CSF2, KLF6, let-7, STAT1, YAP/TAZ	FGF2, HGF, IL1A, IL6, LIN28B, PLAU, PTGS2	Activation of hepatic stellate cells	6.425
mir-193, NOSTRIN	CD151, ERBB4, FLT1, IL6, MAPT, MMP2, NPHS2, PLAU, THBS1	Migration of cells, morbidity or mortality	6
Collagen type IV, IKK, miR-146a-5p, SPARC	ATM, BRCA1, BRCA2, CDH3, CLDN4, CXCL10, FANCA, FANCD2, IL6, MBP, NDRG1, PLAU, PTGS2, RAD54L, TBX2, THBS1, UHRF1	Cell viability	4.608
E2f, E2F3, TFAP2A	BAX, BRCA1, CDC45, CDK1, E2F1, FANCD2, FGF2, KISS1, LIG1, MCM7, THBD, THBS1	Hypoplasia, vascular tumor	3.175

## Data Availability

The data and supportive information are available within the article.

## LIST OF ABBREVIATIONS

BUC: Bladder Urothelial Carcinoma
CCK8: Cell Counting Kit 8
DEGs: Differentially expressed genes
FDR: False Discovery Rate
GEPIA: Gene Expression Profiling Interactive Analysis
GO: Gene Ontology
GSEA: Gene Set Enrichment Analysis
HR: Hazards Ratio
IPA: Ingenuity^®^ Pathway Analysis
IPKB: Ingenuity Pathway Knowledge Base
KEGG: Kyoto Encyclopedia of Genes and Genomes
NES: Normalized Enrichment Score
PPI: Protein-protein Interaction
TCM: Traditional Chinese Medicine

## References

[1] Siegel R.L., Miller K.D., Fuchs H.E., Jemal A. (2021). Cancer statistics, 2021.. CA Cancer J. Clin..

[2] Bray F., Ferlay J., Soerjomataram I., Siegel R.L., Torre L.A., Jemal A. (2018). Global cancer statistics 2018: GLOBOCAN estimates of incidence and mortality worldwide for 36 cancers in 185 countries.. CA Cancer J. Clin..

[3] Jain P., Kathuria H., Momin M. (2021). Clinical therapies and nano drug delivery systems for urinary bladder cancer.. Pharmacol. Ther..

[4] Patel V.G., Oh W.K., Galsky M.D. (2020). Treatment of muscle‐invasive and advanced bladder cancer in 2020.. CA Cancer J. Clin..

[5] SUN D (2022). Inventor method of treating cancer with composition of traditional chinese medicine and its preparation method thereof..

[6] Li X, Xu X, Wang Y (2021). Inventors traditional chinese medicine composition for treating breast cancer and preparation method thereof..

[7] Vitelli Storelli F., Molina A.J., Zamora-Ros R. (2019). Flavonoids and the risk of gastric cancer: An exploratory case-control study in the MCC-spain study.. Nutrients.

[8] Xie Z., Wei Y., Xu J., Lei J., Yu J. (2019). Alkaloids from Piper nigrum synergistically enhanced the effect of paclitaxel against paclitaxel-resistant cervical cancer cells through the downregulation of Mcl-1.. J. Agric. Food Chem..

[9] Zhang M., Zhang Y., Zhang L., Tian Q. (2019). Mushroom polysaccharide lentinan for treating different types of cancers: A review of 12 years clinical studies in China.. Prog. Mol. Biol. Transl. Sci..

[10] Liao Y.H., Li C.I., Lin C.C., Lin J.G., Chiang J.H., Li T.C. (2017). Traditional Chinese medicine as adjunctive therapy improves the long-term survival of lung cancer patients.. J. Cancer Res. Clin. Oncol..

[11] Liu Y., Yang S., Wang K. (2020). Cellular senescence and cancer: Focusing on traditional Chinese medicine and natural products.. Cell Prolif..

[12] Ma X., Yan L., Zhu Q., Shao F. (2017). Puerarin attenuates cisplatin-induced rat nephrotoxicity: The involvement of TLR4/NF-κB signaling pathway.. PLoS One.

[13] Liu X., Li S., Li Y., Cheng B., Tan B., Wang G. (2018). Puerarin inhibits proliferation and induces apoptosis by upregulation of miR-16 in bladder cancer cell line T24.. Oncol. Res..

[14] Xu H., Hu M., Liu M. (2020). Nano-puerarin regulates tumor microenvironment and facilitates chemo- and immunotherapy in murine triple negative breast cancer model.. Biomaterials.

[15] Jiang K., Chen H., Tang K. (2018). Puerarin inhibits bladder cancer cell proliferation through the mTOR/p70S6K signaling pathway.. Oncol. Lett..

[16] Du L., Zhang L., Sun F. (2022). Puerarin inhibits the progression of bladder cancer by regulating circ_0020394/miR-328-3p/NRBP1 Axis.. Cancer Biother. Radiopharm..

[17] Ye G., Kan S., Chen J., Lu X. (2019). Puerarin in inducing apoptosis of bladder cancer cells through inhibiting SIRT1/p53 pathway.. Oncol. Lett..

[18] Zhou Y., Zhou B., Pache L. (2019). Metascape provides a biologist-oriented resource for the analysis of systems-level datasets.. Nat. Commun..

[19] Szklarczyk D., Gable A.L., Lyon D. (2019). STRING v11: Protein–protein association networks with increased coverage, supporting functional discovery in genome-wide experimental datasets.. Nucleic Acids Res..

[20] Tang Z., Li C., Kang B., Gao G., Li C., Zhang Z. (2017). GEPIA: A web server for cancer and normal gene expression profiling and interactive analyses.. Nucleic Acids Res..

[21] Subramanian A., Tamayo P., Mootha V.K. (2005). Gene set enrichment analysis: A knowledge-based approach for interpreting genome-wide expression profiles.. Proc. Natl. Acad. Sci..

[22] Khan M.I. (2016). Dębski KJ, Dabrowski M, Czarnecka AM, Szczylik C. Gene set enrichment analysis and ingenuity pathway analysis of metastatic clear cell renal cell carcinoma cell line.. Am. J. Physiol. Renal Physiol..

[23] Li H., Wang Y., Rong S. (2020). Integrin α1 promotes tumorigenicity and progressive capacity of colorectal cancer.. Int. J. Biol. Sci..

[24] Zheng QH, Li XL, Mei ZG (2017). Efficacy and safety of puerarin injection in curing acute ischemic stroke: A meta-analysis of randomized controlled trials.. Medicine (Baltimore).

[25] Liu B, Tan Y, Wang D (2016). Puerarin for ischaemic stroke.. Cochrane Database Syst Rev..

[26] Cai H., Kondo M., Sandhow L. (2022). Critical role of Lama4 for hematopoiesis regeneration and acute myeloid leukemia progression.. Blood.

[27] Zheng B., Qu J., Ohuchida K. (2020). LAMA4 upregulation is associated with high liver metastasis potential and poor survival outcome of Pancreatic Cancer.. Theranostics.

[28] Wang M., Li C., Liu Y., Wang Z. (2021). Effect of LAMA4 on prognosis and its correlation with immune infiltration in gastric cancer.. BioMed Res. Int..

[29] Li Y., Guan B., Liu J. (2019). MicroRNA-200b is downregulated and suppresses metastasis by targeting LAMA4 in renal cell carcinoma.. EBioMedicine.

[30] Liu Y., Xu Y., Ding L., Yu L., Zhang B., Wei D. (2020). LncRNA MEG3 suppressed the progression of ovarian cancer *via* sponging miR-30e-3p and regulating LAMA4 expression.. Cancer Cell Int..

[31] Deng W.W., Wu L., Bu L.L. (2016). PAK2 promotes migration and proliferation of salivary gland adenoid cystic carcinoma.. Am. J. Transl. Res..

[32] Zhu Z., Song J., Guo Y. (2020). LAMB3 promotes tumour progression through the AKT–FOXO3/4 axis and is transcriptionally regulated by the BRD2/acetylated ELK4 complex in colorectal cancer.. Oncogene.

[33] Moller-Levet C.S., Betts G.N.J., Harris A.L., Homer J.J., West C.M.L., Miller C.J. (2009). Exon array analysis of head and neck cancers identifies a hypoxia related splice variant of LAMA3 associated with a poor prognosis.. PLOS Comput. Biol..

[34] Jones L., Naidoo M., Machado L.R., Anthony K. (2021). The Duchenne muscular dystrophy gene and cancer.. Cell. Oncol..

[35] Tan S., Tan J., Tan S. (2016). Decreased Dp71 expression is associated with gastric adenocarcinoma prognosis.. Oncotarget.

[36] Zhou S., Ouyang W., Zhang X. (2021). UTRN inhibits melanoma growth by suppressing p38 and JNK/c-Jun signaling pathways.. Cancer Cell Int..

