# Supplementary material for: Comprehensive Genomic Analysis of Puerarin in Inhibiting Bladder Urothelial Carcinoma Cell Proliferation and Migration
Source: Recent Pat Anticancer Drug Discov. 2023 Sep 28;19(4):516–29. doi: 10.2174/1574892819666230908110107 (PMC11348475; doi:10.2174/1574892819666230908110107)
Supplement: Supplementary file 1 [file PRA-19-516_SD1.zip › PRA-19-516_SD1/PRA-19-516_SD1.pdf]

SUPPLEMENTARY MATERIAL

Comprehensive Genomic Analysis of Puerarin in Inhibiting Bladder Urothelial Carcinoma Cell Proliferation and Migration

Yu-Yang Ma[1,2,#], Ge-jin Zhang[3,#], Peng-fei Liu[4,5,#], Ying Liu[6,#], Ji-cun Ding[7], Hao Xu[2], Lin Hao[1], Deng Pan[2], Hai-luo Wang[1], Jing-kai Wang[8], Peng Xu[8], Zhen-Duo Shi[1,2,*] and Kun Pang[1,2,*]

[1]Department of Urology, Xuzhou Central Hospital, Xuzhou Clinical School of Xuzhou Medical College. No.199, South Jiefang Road, Xuzhou, Jiangsu, China; [2]Graduate School, Bengbu Medical College, Building 1, Administration Building, 2600 Donghai Avenue, Bengbu City, Anhui Province, China; [3]Department of Urology, Suqian Zhongwu Hospital. No. 3786, Development Avenue, Suqian Economic and Technological Development Zone, Suqian, China; [4]Jiangsu Provincial Key Laboratory of Educational Big Data Science and Engineering, Jiangsu Normal University, 101 Shanghai Road, Tongshan, Xuzhou 221116, China; [5]School of Mathematics and Statistics and Research Institute of Mathematical Sciences (RIMS), Jiangsu Normal University, 101 Shanghai Road, Tongshan, Xuzhou 221116, China; [6]Department of Laboratory, Xuzhou Central Hospital, Xuzhou Clinical School of Xuzhou Medical College. No.199, South Jiefang Road, Xuzhou, Jiangsu, China; [7]Department of Burn and Plastic Surgery, Xuzhou First People's Hospital. No. 269, Daxue Road, Tongshan District, Xuzhou, Jiangsu, China; [8]Graduate School, Jiangsu University, 301 Xuefu Road, Zhenjiang, 212013, Jiangsu Province, China

2212-3970/24 $65.00+.00 **© 2024 Bentham Science Publishers**
